# Age- dependent injury patterns, management and impact on mortality of severe thoracic trauma in severely injured children: a retrospective study from the TraumaRegister DGU^®^

**DOI:** 10.1186/s13049-025-01510-3

**Published:** 2025-11-15

**Authors:** Ina Fichtel, Sophie Fleischer, Lisa Bode, Rolf Lefering, Ferdinand C. Wagner, Katrin Fink, Paula Beck, Hagen Schmal, Jörg Bayer

**Affiliations:** 1 Department of Trauma and Orthopaedic Surgery, Schwarzwald-Baar Hospital, Klinikstraße 11, 78052 Villingen-Schwenningen, Germany; 2 Department of Orthopaedic and Trauma Surgery, Medical Center, Faculty of Medicine, University of Freiburg, Hugstetter Straße 55, 79106 Freiburg, Germany; 3 Institute for Research in Operative Medicine (IFOM), Faculty of Health, University Witten/Herdecke, Ostmerheimer Straße 200, 51109 Cologne, Germany; 4 Faculty of Medicine, University Emergency Center, Medical Center, University of Freiburg, Sir-Hans-A.-Krebs-Straße, 79106 Freiburg, Germany; 5 Department of Orthopedic Surgery, University Hospital Odense, Sdr. Boulevard 29, Odense, 5000 Denmark

**Keywords:** Pediatric, Polytrauma, Multiple injuries, Thoracic trauma, Trauma management, Outcome, Mortality

## Abstract

**Background:**

Severe thoracic trauma in children is rare and often underestimated. While standardized protocols exist, the rarity of polytrauma in children may lead to uncertainties in their application. Thus, we aim to characterize severely injured children with significant thoracic trauma and identify age-dependent differences in prehospital and early clinical management.

**Methods:**

Patients documented by German hospitals between 2008 and 2023 in the TraumaRegister DGU^®^, aged ≤ 20 years and sustaining at least serious chest injury (AIS_Thorax_ ≥ 3) were analyzed. Patients were grouped by age: 0–5 years, 6–12 years and 13–16 years and statistically compared; additional data from patients aged 17–20 years is provided. Demographic, clinical characteristics and treatment comparing the aforementioned groups were evaluated using descriptive statistics. In the group of 0–16 years old independent risk factors for mortality were scrutinized applying multiple logistic regression analysis.

**Results:**

A total of 5,040 severely injured patients were analyzed (310 patients aged 0–5 years, 475 aged 6–12 years, 984 aged 13–16 years and 3,271 in group 17–20 years). With increasing age, significantly more males were injured and mechanism of injury was significantly different between the age groups. During prehospital management significant age dependent differences were seen regarding helicopter emergency medical service treatment, intubation, chest tube placement, application of catecholamines or tranexamic acid. Significant differences were found in terms of injured body regions, where the youngest suffered most from leading thoracic injury and injury to the head while abdominal injury and injury to extremities happened significantly more often in the 13–16 years old. During treatment the 13–16 years old received more blood products, were more likely to undergo thoracic surgery and stayed longer on the respective wards. Multivariate logistic regression showed an independent association with a significant mortality risk for MAIS ≥ 4 (OR = 2.87; *p* = 0.03), polytrauma (OR = 3.09; *p* < 0.001) and the need for blood transfusion before admission to the intensive care unit (OR = 2.46; *p* < 0.001).

**Conclusions:**

Treating severely injured children is always challenging, even more so when they have suffered critical trauma to the chest. With our results we offer starting points for age-dependent injury prevention and provide information to analyze and question current (pre-) hospital management protocols.

## Background

Severe thoracic trauma in children is rare and may be underestimated [1–3]. Additionally, severe thoracic trauma is associated with increased mortality [4]. The percentage of German pediatric patients with major trauma suffering from associated significant chest injuries is age-dependently increasing and can be as high as 44% in 11- to 15-year-old children [5]. The most common diagnosis in pediatric thoracic trauma is lung contusion in combination with hemato-/pneumothorax and/or rib fractures [6]. In former studies, regression analysis in pediatric patients with single-system injury revealed only Abbreviated Injury Scale (AIS) for head and chest trauma to be associated with increased odds for mortality. Additionally, mortality for chest injuries consistently increases across AIS scores in both single-system and multi-system injuries [7]. Anatomical differences, such as greater chest wall flexibility, a thinner thoracic wall, and a relatively larger mediastinum, can make even severe injuries appear initially inconspicuous [6], increasing the risk of delayed diagnosis and treatment. Furthermore, respiratory and circulatory physiology differ across pediatric age groups [8]. Children have anatomic, physiologic, psychological, hemostatic and epidemiologic particularities mandating pediatric-specific therapeutic protocols and interventions [6, 9, 10]. A wide range of procedures may become necessary during prehospital and trauma room treatment of severely injured children. Endotracheal intubation might be necessary to secure the airway and sustain oxygenation, as well as chest tube insertion and hemodynamic stabilization [4, 11, 12]. While standardized protocols for pediatric trauma exist, the rarity of polytrauma in children may lead to uncertainties in their application [13] which emphasizes the importance of theoretical education and practical training programs for appropriate management of pediatric trauma and primary survey or resuscitation as well as early induction of adequate therapy of severely injured children [14]. Therefore, knowing what to expect when treating severely injured children with significant thoracic injury and regular team training in pediatric polytrauma care is mandatory. A trauma center with an organized training system and certification can show better results in trauma treatment [15]. Thoracic trauma management in severely injured adults has been studied [16, 17], but the specific challenges of pediatric thoracic trauma in severely injured children require further investigation. In the emergency setting, timely and accurate risk assessment can be challenging, particularly for providers with limited experience in pediatric trauma care.

Thus, in this study we aim to characterize severely injured children with significant thoracic trauma and identify differences in preclinical and early clinical management depending on age and compare their treatment to younger adults. With this information, we aim to enrich our knowledge on what to expect when treating severely injured children with serious chest injuries and provide information to evaluate current management protocols.

## Methods

The TraumaRegister DGU^®^ (TR-DGU) of the German Trauma Society (Deutsche Gesellschaft für Unfallchirurgie, DGU) was founded in 1993 and represents a multi-center database containing pseudonymized and standardized documentation of severely injured patients. Data are collected prospectively in 4 consecutive phases: (A) pre-hospital phase, (B) trauma room and initial surgery, (C) intensive care unit and (D) discharge. The documentation includes detailed information on demographics, injury patterns, comorbidities, pre-hospital and clinical management, intensive care course, key laboratory findings including transfusion data, and outcome. The inclusion criterion is admission to hospital via the trauma room with subsequent monitoring in intensive or intermediate care, or arrival at hospital with vital signs and death before admission to intensive care unit (ICU).

The infrastructure for documentation, data management and data analysis are provided by the AUC - Academy for Trauma Surgery (AUC - Akademie der Unfallchirurgie GmbH), which is affiliated to the German Trauma Society. The scientific leadership is provided by the Committee on Emergency Medicine, Intensive Care and Trauma Management (Sektion NIS) of the German Trauma Society. Participating clinics enter their pseudonymized data into a central database via a web-based application. Scientific analyses are approved according to a peer review process defined in the publication guidelines of the TR-DGU. The participating clinics are mainly located in Germany (90%), but an increasing number of clinics from other countries are also contributing data (currently from Austria, Belgium, China, Finland, Luxembourg, Slovenia, Switzerland, the Netherlands and the United Arab Emirates). Currently, about 38,000 cases from almost 700 clinics are added to the database each year. Participation in the TR-DGU is on a voluntary basis. It is mandatory for TraumaNetzwerk DGU^®^ clinics to enter at least one basic data set for quality assurance purposes.

In Germany, every certified trauma center can provide at least the initial treatment for injured children but, usually, children are directly brought to a designated level 1 trauma center.

The documentation of a basic dataset in the TR-DGU had been introduced by the German Trauma Society when the registry became obligatory for the certification as a trauma center. The basic data collection form is mainly used by smaller hospitals and covers only about 50% of the data collected in the more extensive standard data collection form. Since relevant information used in this analysis is available in the standard dataset only (e.g. chest tube), cases with reduced documentation by basic data collection form had to be excluded.

The study strictly adheres to the publication guidelines of the TR-DGU and is registered under the TR-DGU project ID 2022-025. The research project has been approved by the local ethics committee (University of Freiburg, 24-1586-S1-retro).

### Patients

This retrospective multicenter observational study evaluated the TR-DGU dataset statistically to extract relevant data on all severely injured children, adolescents and young adults ≤ 20 years old admitted to a German trauma center from 01.01.2008 to 31.12.2023 with an at least serious chest injury defined as AIS_Thorax_ ≥ 3.

Patients transferred in from another hospital, and patients transferred out early (within 48 h after admission) to another facility were excluded. Also, patients documented with the reduced basic dataset were excluded.

Injuries were graded according to the 2005, update 2008, version of the Abbreviated Injury Scale (AIS) [18], and the ISS was calculated as described earlier by Baker et al. [19]

Thoracic injuries are those coded as AIS = 4xxxxx.x not including the thoracic spine [20]. An injury was considered “serious” if the AIS severity was at least 3 points.

Maximum AIS (MAIS) is defined as the highest AIS score assigned to any single injury a patient has sustained, thus reflecting the severity of the worst injury.

We defined a polytraumatized patient according to the “Berlin definition”, which is a patient suffering from significant injuries (AIS ≥ 3) in two or more different anatomic AIS regions in conjunction with at least one defined pathophysiologic parameter [21]. 

Length of hospital stay was defined as the number of days from admission to discharge or death. Multiple organ failure (MOF) was defined as failure of ≥ 2 organs for ≥ 2 days and sepsis was assessed as previously published [22, 23]. Sepsis was defined as a systemic response to infection, evidenced by the presence of microorganisms. Data on respiratory failure, MOF, and sepsis were available for patients documented via the standard data collection form. Blood transfusion is defined as administration of at least one unit of packed red blood cells prior to ICU admission (i.e. during stay in the emergency department or during initial surgery). Chest tube placement refers to any kind of pleura decompression (e.g. needle, chest tube, etc.). Emergency thoracotomy relates to a thoracotomy performed prior to ICU admission (either in the emergency department or operating room). Thoracic surgery comprises any kind of surgical intervention to treat a thoracic injury.

### Patient subgroups

For further analysis, patients were grouped based on their age as follows: 0–5 years, 6–12 years and 13–16 years; for comparison, patients aged 17–20 years served as a corresponding group similar to adults.

### Statistical analysis

Demographic and clinical characteristics comparing the groups were evaluated using descriptive statistics. Continuous variables are presented as mean with standard deviation (SD) or median with quartiles (Q), as appropriate, while categorical variables are presented as number of cases (n) with percentages (%). The respective statistics refer to patients with valid data sets only. Therefore, the total number of patients or characteristics for analysis may vary.

Statistical comparison of subgroups was performed for the three children subgroups only. Results in young adults are added for descriptive reasons only. Categories were compared with a chi-squared test; metric values with Kruskal-Wallis test. A p-value < 0.05 is considered statistically significant. Pairwise post-hoc tests were avoided to limit the number of statistical tests and even the presented p-values require careful interpretation.

Statistical analysis of mortality was performed using multiple logistic regression analysis (MLR), with hospital mortality as dependent variable. Only children (aged 0–16 years) were included. The independent variables consisted of age, gender, MAIS, polytrauma, head injury, abdominal injury, extremity injury, blood transfusion, unconsciousness (GCS 3–8), cardiopulmonary resuscitation and type of thoracic injury (rib fractures, lung contusion, hemothorax, pneumothorax, vascular injury). Prehospital shock, defined as SBP ≤ 90mmHg, is a known prognostic factor and is often used as an indirect sign of relevant bleeding. Although shock may occur for other reasons, in trauma it is highly correlated with blood loss. In our predictive model, we used the need for blood transfusion as an indirect sign of relevant bleeding.

Statistical analysis was conducted using SPSS (Version 29, IBM Inc., Armonk, NY, USA).

## Results

5,832 patients in the database met the inclusion criteria. After excluding patients being transferred out within 48 h (*n* = 169, 2.9%; since their final outcome was missing) and excluding transferred in patients after pre-treatment in another hospital (*n* = 623, 10.7%; because their initial treatment documentation was missing), a total of 5,040 severely injured patients with relevant thoracic trauma were identified for further analysis. Datasets were documented by 205 German hospitals. There were 310 patients in the age group 0- to 5-years, 475 in the age group 6- to 12-years and 984 patients in the age group 13- to 16-years. The age group of young adults aged 17- to 20-years included 3,271 patients and served as a group for comparison. Data analyzed represents patients treated in Level-1 (86.5%), Level-2 (11.7%) and Level-3 (1.7%) trauma centers, respectively, but only documented by the extensive standard data collection form.

### Demographics

Injured children were predominately male in all subgroups. Most patients sustained their injuries in traffic accidents. The percentage of car accidents is highest in the young adults (41.9%) and almost unchanged in the different age groups of 0- to 16-year-olds (12.3% − 13.5%). While motorcycle accidents play a minor role among children aged 0- to 12-years (0.2% − 1.6%), this injury mechanism surges in the 13- to 16-years-old age group (24.2%). Falls from > 3 m height occurred predominately in the age group of 0- to 5-year-olds, while injured pedestrians were most commonly found in the 6- to 12-years-old group. Bicycle accidents occurred mainly in the age groups of 6- to 16-year-olds. The study group basic distribution of injury mechanisms and significant differences is summarized in Table 1.

**Table 1:** Demographics and injury mechanism

Age group (years)	0–5 *n* = 310	6–12 *n* = 475	13–16 *n* = 984	*p*-value	17–20 *n* = 3271
Males (%)	60.0	62.2	68.6	**0.005**	77.1
**Mechanism**					
Traffic (%)	47.4	72.8	69.7	**< 0.001**	78.4
Car (%)	13.6	12.3	13.5	0.362	41.9
Motorcycle (%)	0	1.1	26.3	**< 0.001**	21.7
Bicycle (%)	3.4	14.3	13.1	**< 0.001**	2.8
Pedestrian (%)	22.4	34.7	10.1	**< 0.001**	4.0
Fall > 3 m (%)	36.2	14.3	17.2	**< 0.001**	10.8
Fall < 3 m (%)	4.2	4.1	3.2	0.586	1.4

### Prehospital management

Endotracheal intubation was performed in 45.7% − 53.3% of the patients before reaching the hospital. Chest tubes were placed overall in 8.1% of patients, ranging from 5% in the 0- to 5-years-olds to 9% in older children. The highest rate of measured systolic blood pressure (SBP) ≤ 90mmHg is documented in the youngest age group with 25.6%. In the other age groups a SBP ≤ 90mmHg is consistently documented in about 19% of severely injured children. The reported SBP < 90mmHg in 0–5 years old might not represent hemorrhagic shock in infants but might be surrogated and supported by prehospital catecholamine administration in 14.7% and an early hospital transfusion rate of 18.6% in this age group. The application of catecholamines and tranexamic acid is predominantly found in the group of 13- to 16-years-old with 18.7% and 27.2%, respectively. Comprehensive data on prehospital interventions are shown in Table 2.

**Table 2:** Prehospital data

Age group (years)	0–5 *n* = 310	6–12 *n* = 475	13–16 *n* = 984	*p*-value	17–20 *n* = 3271
Level of consciousness (GCS 3–8) (%)	39.0	33.0	39.0	0.085	33.4
Intubation (%)	45.7	47.0	53.3	.**017**	49.4
Chest tube (%)	5.0	4.9	9.0	.**006**	8.7
Catecholamines (%)	14.7	13.3	18.7	.**024**	15.7
Tranexamic Acid (%)	6.3	17.0	27.2	**< 0.001**	26.4
Shock (SBP ≤ 90 mmHg) (%)	25.6	19.0	19.4	0.113	19.4
HEMS	38.1	48.3	42.8	**0.018**	37.6

Percentages of patients within the respective age group receiving the indicated prehospital intervention

For better assessment of intubation rates the percentage of reduced level of consciousness (GCS 3–8) for each age group is shown

Statistical comparison of subgroups was performed for three subgroups (0–16 years) only. Results in young adults (17–20 years) are added for descriptive reasons only

GCS = Glasgow Coma Scale; HEMS = transport by helicopter; SBP = systolic blood pressure

### Clinical management

A computed tomography scan (CT) of any body region was performed in > 88% of the documented severely injured children, while > 80% received a whole-body computed tomography scan (WB-CT), the use of any of these diagnostic tools was increasing with age. A plain X-ray of any kind was taken in > 33% and focused abdominal ultrasound (FAST) was used as a diagnostic tool in > 89% of the patients. Only rates of applied WB-CT and FAST showed significant differences between the age groups, with FAST-protocols being applied more often in younger and WB-CT in older age groups.

Upon hospital admission 14.6% − 28.2% of patients presented with systolic blood pressure (SBP) ≤ 90mmHg with significantly more patients in shock in younger ages. Mean base excess (BE) was determined to be -3.6 to -5.2 mmol/l with significantly lower numbers in younger children (Table 3).

**Table 3:** Clinical diagnostics and treatment

Age group (years)	0–5 *n* = 310	6–12 *n* = 475	13–16 *n* = 984	*p*-value	17–20 *n* = 3271
CT (%)	89.7	88.4	92.1	0.064	93.4
Whole-body CT (%)	80.8	81.8	88.0	**< 0.001**	90.5
X-ray (%)	33.9	35.5	37.8	0.398	37.3
Abdominal Ultrasound (%)	92.5	93.4	89.1	**0.015**	89.3
Shock (SBP ≤ 90 mmHg) (%)	28.2	16	14.6	**< 0.001**	15
BE mean (± SD) mmol/l	-5.2 (6.4)	-3.7 (5.5)	-3.6 (5.9)	**< 0.001**	-3.2 (5.9)
Blood Transfusion (%)	18.6	17.5	23.5	**0.016**	19.8
ICU Admission (%)	91.0	91.8	91.9	0.878	91.5
MV on ICU (%)	61.7	57.6	62.9	0.165	62.3
Sepsis (%)	4.1	4.0	5.0	0.677	5.4
MOF (%)	19.2	20.1	24.3	0.098	24.4
Thoracic Surgery (%)	21.6	19.4	29.8	**< 0.001**	34.5
Emergency Thoracotomy (%)	4.0	1.6	4.7	0.105	4.3
LOMV (days) median [Q]	1 [0–4]	1 [0–3]	1 [0–4]	0.088	1 [0–5]
LOICUS (days) median [Q]	4 [1–10]	3 [1–8]	4 [1–11]	**0.039**	4 [1–12]
LOHS (days) median [Q]	9 [5–18]	10 [5–17]	13 [6–21]	**0.001**	13 [6–22]

Early clinical parameters, diagnostics, emergency and intensive care united treatment

Initial rates of SBP ≤ 90mmHg and BE measurements in the ED are shown

Rates of CT (any body region) and whole-body CT scans, X-ray (any body region) and focused abdominal sonography applications are depicted

Further treatment with ICU admission rates, mechanical ventilation, incidence of sepsis and MOF, and thoracic surgeries are shown

Emergency thoracotomy is a subset of thoracic surgery

Statistical comparison of subgroups was performed for three subgroups (0–16 years) only. Results in young adults (17–20 years) are added for descriptive reasons only

CT = computed tomography; BE = base excess; ICU = intensive care unit; LOHS = length of hospital stay; LOICUS = length of ICU stay; LOMV = length of mechanical ventilation; MOF = multiple organ failure; MV = mechanical ventilation; Q = quartiles; SBP = systolic blood pressure; SD: standard deviation

Emergency procedures before ICU admission included administration of packed red blood cells (PRBC), which was required in > 17.5% of the severely injured children to counter hemorrhagic shock. In our collective > 4% of the pediatric patients underwent an emergency thoracotomy and > 19% required thoracic surgery. While rates of emergency thoracotomy are almost equally distributed, necessity in thoracic surgery increases with age and culminates in almost 30% in the adolescents, compared to 34.5% in young adults. Chest tubes were placed in 23.1% of our patients in the emergency department (ED), with 176 patients having already received a chest tube prehospitally. The rate of patients admitted to the ICU with a chest tube placed either prehospitally or in the ED was 27.7%.

While similar rates of children in each age group were referred to the intensive care unit for further treatment, also almost equal percentages required mechanical ventilation (> 57%) irrespective their according age group. In contrast, with increasing age sepsis rates and multiple organ failure (MOF) increased, peaking at 5% and 24.3%, respectively, in the adolescents. There were neither statistically significant differences in sepsis and MOF rates between the adolescents and younger children nor were they different to young adults.

With respect to mechanical ventilation, the median time spent on a respirator was 1 day for all age groups. Statistically significant differences exist between the age groups in length of ICU and hospital stays, with a median of 3–4 days and 9–13 days, respectively and the eldest staying the longest, in ICU and hospital. Only length of hospital stay was significantly different between the age groups.

Procedures performed during early clinical management and patient stabilization in the emergency department and intensive care unit are listed in Table 3.

### Injuries

The distribution of relevant (AIS ≥ 3) injuries to the respective body regions within each age group is listed in Table 4. In about 37.0% − 44.2% over all age groups the thoracic trauma is the leading injury, representing the AIS_Thorax_ is higher than the AIS_Head_, AIS_Extremities_ and/or AIS_Abdomen_. The percentage of patients with at least serious injuries to the head (AIS_Head_ ≥3) was highest in the youngest age group (48.4%). The frequency of a relevant abdominal injury (AIS_Abdomen_ ≥3) was highest in the 13- to 16-years-old (24.2%) and lowest in the youngest children (14.2%). Relevant injuries to the extremities (AIS_Extremities_ ≥3) are unequally distributed, occurring the least often in the group of 0- to 5-year-olds (18.4%). The mean ISS is similar in all age groups, ranging between 27.1 and 30.7. Rates of injured body regions and the ISS significantly differ between the respective age groups.

**Table 4:** Injury severity

Age group (years)	0–5 *n* = 310	6–12 *n* = 475	13–16 *n* = 984	*p*-value	17–20 *n* = 3271
Head injury AIS ≥ 3 (%)	48.4	39.4	43.5	**0.043**	38.3
Abdominal injury AIS ≥ 3 (%)	14.2	17.3	24.2	**< 0.001**	21.7
Extremity injury AIS ≥ 3 (%)	18.4	28.0	33.9	**< 0.001**	37.0
Leading thoracic trauma (%)	44.2	41.9	37.0	**0.037**	40.4
Injury Severity Score (mean, SD)	28.8 (14.9)	27.1 (13.4)	30.7 (14.9)	**< 0.001**	29.1 (14.6)

Distribution of relevant (AIS ≥ 3) injuries to respective body regions and overall mean ISS per age group

Statistical comparison of subgroups was performed for three subgroups (0–16 years) only. Results in young adults (17–20 years) are added for descriptive reasons only

Leading Thoracic trauma = AIS_Thorax_ is higher than the AIS_Head_, AIS_Extremities_ and/or AIS_Abdomen_

SD = standard deviation

Distribution of relevant (AIS ≥ 3) injuries to respective body regions and overall mean ISS per age group

Statistical comparison of subgroups was performed for three subgroups (0–16 years) only. Results in young adults (17–20 years) are added for descriptive reasons only

Leading Thoracic trauma = AIS_Thorax_ is higher than the AIS_Head_, AIS_Extremities_ and/or AIS_Abdomen_

SD = standard deviation

The types of thoracic injuries in severely injured children with AIS_Thorax_ ≥3 is shown in Table 5. Pulmonary contusion is the most common thoracic injury across all age groups (71% − 80%), followed by pneumothorax (32% − 47%) and rib fractures (27% − 39%). A hemothorax is found in 11% − 19%. While the rate of lung contusion decreases in older children, the rate of rib fractures, pneumo- and hemothorax increases. For these stated injuries statistically significant differences exist between the age groups.

**Table 5:** Thoracic injuries

Age group (years)	0–5 *n* = 310	6–12 *n* = 475	13–16 *n* = 984	*p*-value	17–20 *n* = 3271
Scapula fracture (%)	2.9	4.2	10.0	**< 0.001**	9.4
Clavicle fracture (%)	9.4	9.3	12.6	0.09	13.1
Sternum fracture (%)	2.6	1.9	4.3	**0.044**	7.7
Rib fracture (%)	26.8	31.4	35.9	**0.008**	38.7
Hemothorax (%)	11.0	12.2	18.2	**< 0.001**	18.6
Pneumothorax (%)	37.4	31.8	45.4	**< 0.001**	46.9
Lung injury (%)	6.5	8.0	10.3	0.084	8.2
Lung contusion (%)	76.5	79.6	71.6	**0.003**	71.3
Thoracic vessel (%)	0.6	0.4	2.7	**0.002**	3.4

Characterization and rates of thoracic injuries within distinct age groups

Classification according to the AIS codes: parenchymal injuries were summarized as lung injuries while contusions were grouped separately as lung contusions

Statistical comparison of subgroups was performed for three subgroups (0–16 years) only. Results in young adults (17–20 years) are added for descriptive reasons only

Specific injury to the lung had a consistently low prevalence with 7% to 10% in all age groups. Cardiac injuries were observed in 3.7% − 6.3% of cases, primarily consisting of cardiac contusions (AIS = 1). Specific thoracic vascular injuries are very rare (< 1%) in the younger age groups from 0 to 12 years and occur in 3% in the older age groups 13- to 20-years-old.

The number of surgical interventions increases significantly with increasing AIS_Thorax_. Associated clavicle fractures with severe thoracic injury were observed in 9% in the age group 0- to 12-years-old and 13% in the age group 13- to 20-years old. The prevalence of scapula fractures was 2.9% and 4.2% in the younger age groups, but more than doubled in the older age groups (9.4% and 10%, respectively). A similar pattern was found for sternal fractures with 2.6% and 1.9% in younger age groups versus 4.3% and 7.7% in the adolescents and young adults.

### Outcome

Mortality rate was highest in the age group 13- to 16-years-old (16.9%), followed by the age group of 0- to 5-years (15.5%), 6- to 12-years (13.1%) and lowest in the young adults aged 17- to 20-years (12.4%). A significant number of patients (9.1%-13.2%) died within the first 24 h after hospital admission.

### Analysis of mortality risk factors

A logistic regression analysis identified key factors significantly associated with mortality. Table 6 illustrates the odds ratios of predictors of mortality identified in the logistic regression analysis. The mortality risk independently increases significantly with injury severity. Compared to AIS = 3 (reference category), patients with MAIS = 4 in any region had an OR of 2.87 (*p* = 0.03), MAIS = 5 in any region an OR of 14.58 (*p* < 0.001), indicating a higher probability of fatal outcome. Patients presenting with significantly impaired consciousness (GCS ≤ 8) had a 10-fold increase in mortality risk (OR = 10.11; *p* < 0.001). Patients requiring prehospital cardiopulmonary resuscitation (CPR) had an OR of 13.61 (*p* < 0.001) for mortality.

**Table 6:** Multivariate logistic regression analysis in children (age 0–16 years) with hospital mortality as dependent variable

Predictor	OR	95% CI for OR	*p*-value
**Age (reference: 0–5 years)**			0.37
6–12 13–16	1.18 1.50	0.59–2.36 0.81–2.77	0.65 0.20
**Male gender**	0.85	0.54–1.34	0.49
**MAIS (reference AIS = 3)**			**< 0.001**
MAIS = 4 MAIS = 5/6	2.87 15.3	1.09–7.57 6.1–38.6	**0.03** **< 0.001**
**Head injury (AIS ≥ 3)**	1.11	0.60–2.07	0.74
**Abdominal injury (AIS ≥ 3)**	0.72	0.43–1.20	0.20
**Extremity injury (AIS ≥ 3)**	1.41	0.87–2.27	0.16
**Polytrauma (Berlin definition)**	3.09	1.79–5.33	**< 0.001**
**Consciousness level (GCS 3–8)**	10.34	5.17–20.69	**< 0.001**
**Prehospital CPR**	14.31	8.04–25.48	**< 0.001**
**Blood transfusion (before ICU)**	2.46	1.55–3.92	**< 0.001**
**Thoracic injuries**			
**Rib fracture**	0.93	0.57–1.50	0.75
**Lung contusion**	0.65	0.39–1.06	0.086
**Hemothorax**	1.46	0.81–2.62	0.21
**Pneumothorax**	0.96	0.59–1.54	0.85
**Thoracic vessel injury**	0.70	0.18–2.70	0.61

Odds ratios of predictors of mortality identified in the logistic regression analysis. Higher values indicate a stronger association with mortality risk

Total patients *n* = 1,605

AIS = Abbreviated Injury Scale; CPR = cardiopulmonary resuscitation; GCS = Glasgow Coma Scale; ICU = intensive care unit; MAIS = maximum AIS; OR = odds ratio; Polytrauma is defined by the “Berlin definition” [21]

Polytraumatized children with accompanying significant thoracic trauma had a 3-fold increase in mortality risk (OR = 3.1; *p* < 0.001). The necessity for packed red blood cell transfusion (PRBC) in the emergency department and first surgery before ICU admission was also significantly associated with mortality (OR = 2.47; *p* < 0.001).

Although no specific single thoracic injury significantly increased the risk of mortality itself, hemothorax was found to be the entity with the highest associated mortality risk.

## Discussion

We present data of 5,040 retrospectively analyzed severely injured children with, at least, an accompanying serious thoracic trauma (AIS_Thorax_ ≥ 3). The patients were divided by age into four subgroups (0–5, 6–12, 13–16 and 17–20 years, respectively) and differences in the mechanism of injury, injury patterns, prehospital management, clinical care, and outcome are reported.

### Demographic differences and injury mechanisms

Consistent with previous findings in the literature [1, 3, 5, 24], we report an increasing percentage of males constituting the injured with increasing age. The type of injury mechanism varied by age and is an expression of higher mobility levels in the adolescent. While younger children were more frequently involved in falls and pedestrian accidents, traffic accidents dominated injury mechanisms in older patients. This has also been shown in various studies before [3, 5, 6, 25, 26]. 

### Prehospital management

Prehospital interventions - such as endotracheal intubation, chest tube insertions, administration of catecholamines and tranexamic acid (TXA) were performed at different rates between the age groups.

Notably, in the 0- to 5- and 6- to 12-year-old age group only 5% received a chest tube prehospitally, whereas in the older group this intervention was performed almost twice as often (9%). These differences may reflect age-dependent variations in anatomy, injury patterns and clinical decision-making, as suggested before [9, 27, 28]. In a comparable study of polytraumatized children with a median ISS of 24 and 70% thoracic trauma (AIS_Thorax_ ≥2) chest tubes were placed between 11% − 21% in different age groups. Most thoracic drainages were placed in children older than 11 years [26]. In their study, Schuster et al. reported on patients already admitted to the hospital and, thus, interventions undertaken in the hospital. Störmann et al. published pre- and in-hospital chest tube placement in polytraumatized children with thoracic injury. They found overall 28.3% of their patients suffering from thoracic injury needed thoracic drainage, but 62.5% of all chest tubes were placed in the ED, after hospital admission [1]. Additionally, Wyen et al. reported placement of chest tubes in severely injured pediatric patients younger than 17 years prehospitally in 2.5% − 4.8%, but in the ED in up to 15.3% in teenagers [29]. This corresponds to our prehospital data with overall chest tube insertions in 27.7%, with 23.1% placed in the ED.

TXA is widely used in adult trauma care as an antifibrinolytic agent to reduce blood loss and improve hemodynamic stability. However, its role in pediatric trauma remains controversial [30, 31]. While TXA has shown benefits in adult trauma patients, its efficacy and optimal dosing in children is less established [10, 31]. This might be reflected by our observed rates of inconsistent TXA administration, especially in children younger than 5 years of age and 6- to 12-years, despite comparable use of catecholamines and prevalence of shock in our collective. Younger children may have received TXA less frequently due to a lack of standardized protocols, concerns about age-specific pharmacokinetics, and the assumption that pediatric patients exhibit better physiological compensation for blood loss.

The priority during initial assessment of children with thoracic trauma should be the airway, its patency and whether or not there are respiratory movements [32] and adequate oxygenation. We found age dependent prehospital intubation rates of 45% − 53%, with the lowest rate in 0- to 5-year-old and the highest in 13- to 16-year-old children. This is considerably lower than published intubation rates of >71.6% by Schuster et al. or 74.3% by Störmann et al. [1, 26] but comparable to reports of 41% − 61% in pediatric patients aged 17 and younger by Wyen et al. [29] Interestingly, Wyen et al. evaluated TR-DGU data from 1993 to 2007, which constituted the 15-year period preceding our study, in multiply-injured patients and reported on children ≤ 17 years of age with mean ISS ranging from 21.0 (infants) to 26.7 (teenagers). In this context our population had higher ISS values and suffered from at least serious chest trauma, yet prehospital intubation rates were not increased.

### Clinical diagnostics and management

Clinical measures, such as limited CT scans, WB-CT, and X-ray imaging, were performed very frequently across all age groups, with an increase in CT usage with age. In our study we documented selective CT and WB-CT rates of 80% − 93%, which appear to be high and must be discussed. The true extent of the benefits of whole-body CT has been questioned precisely, because the importance of additional findings and the implications for clinical findings are still debated as they may have little impact on patient management or prognosis [33]. Computed tomography should be carried out in children in accordance with defined criteria, as a team decision and with the use of age-specific low-dose CT protocols (ALARA, as low as reasonably achievable, principle) [34, 35]. This is why continuing efforts are undertaken to optimize the use of WB-CT in injured children [36]. 

Due to our patient selection, we had a >90% admission rate on the ICU for all age groups. Yet, in comparison to Buschmann et al. who studied multiply injured children characterized by a comparable injury severity (ISS 26.4–29.6) their children were longer mechanically ventilated (median 2 days), stayed longer on ICU (median 5-7.5 days) and in the hospital (median 13–18 days) [5]. Nevertheless, Buschmann et al. investigated patient data from 1997 to 2003, and, in this context, medical treatment and ICU principles have evolved over ensuing years, which contributed to the fact that our ICU and airway management data have improved in comparison. On the other hand, our pediatric patients had higher rates of mechanical ventilation (58%-62% vs. 25.6%) and emergency thoracotomy (1.6%-4.7% vs. 0.6%) compared to Jung et al. [15] However, in our collective, the rate of thoracic operations and emergency thoracotomies increased with age, which may reflect the occurrence of more complex thoracic injuries, especially hemothorax and injury to thoracic vessels in older patients. Another explanation for our high rates of thoracic surgery, including emergency thoracotomy, might be documentation of chest drain insertion via mini-thoracotomy as thoracic surgery.

### Injury patterns

In our study we report on age related injury mechanisms that mirror age dependent degrees of mobility and risk behavior. Falls and passive involvement in traffic accidents in the youngest shifts towards accidents due to active participation in traffic and utilization of high velocity motor vehicles generating high energy trauma in adolescents. This is comparable to frequent studies on trauma in childhood and adolescence [6, 26, 37]. These findings could be used to identify age-specific prevention strategies that e.g. focus particularly on road safety in the adolescents [37]. 

The distribution of injuries differed considerably between the age groups in our patients. Severe head injuries occurred most frequently in the 0– to 5-year-old group, whereas extremity and abdominal injuries increased in older patients. These injury patterns have been documented before [2, 5, 29]. Uniquely, we report on severely injured children and adolescents with at least serious thoracic injuries. In polytraumatized children head and thoracic trauma are most prevalent [5, 26, 37] and thoracic trauma is a relevant factor for emergency interventions, morbidity and mortality [4, 7]. In our collective the youngest children had the highest incidence of leading thoracic trauma, with AIS_Thorax_ representing the highest AIS value of all sustained injuries. Yet, this finding did not result in significantly higher rates of prehospital intubation, emergency thoracotomy, thoracic surgery or mechanical ventilation on ICU compared to children of older ages or adolescents. One reason might be the distribution of injuries within the thorax. The increasing incidence of rib fractures, hemothorax, and vascular injuries with age may reflect not only a higher exposure to high-energy trauma mechanisms in adolescents and young adults, but also age-related differences in thoracic wall compliance. Younger children have a more elastic ribcage, which can absorb energy without fracturing, leading to a higher incidence of parenchymal injuries such as pulmonary contusions rather than bony injuries [4, 10, 15]. In contrast, older adolescents exhibit a more rigid thoracic cage, making them more susceptible to rib fractures and associated complications such as hemothorax. This, again, is a condition often requiring thoracic surgery [4]. In our collective, we found 2.6% sternum fractures and 27% rib fractures in our youngest age group, which is debatably high since an increased compliance of children’s ribs allows the ribs to bend without fracturing [9]. On the other hand, our mean ISS in this age group was 28.8 and thus represents multiple injuries due to a significant trauma mechanism and impact. Overall, our prevalence of rib and sternal fracture is comparable to previously published data of 31% and 4.2%, respectively [15]. 

We only had a limited number of cardiac injuries (3.7%-6.3%) in our patients, primarily consisting of cardiac contusion. Thoracic vascular injuries occured even less frequently in younger children (< 1%) but increased to 3% in adolescents and young adults. This is consistent with previously published literature [1, 15, 38]. 

### Outcome and mortality risk factors

We found age dependent mortality rates between 12.4% and 16.9%, with the highest rates in the age groups of 0- to 5- and 13- to 16-years, 15.5% and 16.9%, respectively. This is comparable to previously published mortality rates in severely injured children. Price et al. reported a 30-day mortality rate in pediatric trauma patients with ISS ≥ 16 of 12% overall, in 0- to 4-years-old 16%, in 5- to 8-years-old 14%, in 9- to 13-years-old 17% and in 14- to 17-years-old 8% [37]. With a comparable ISS of 24–30 Schuster et al. published 30-day mortality rates of 29.2% in the 0- to 5-years-old, 14.8% in 6- to 10-years-old, 23.7% in 11- to 15-years-old and 15.8% in the 16- to 18-years-old age groups [26]. These age patterns show higher mortality rates in the youngest age groups compared to lower rates in adolescents. This is interesting, since our multivariate logistic regression analysis did not reveal a significant independent mortality risk reduction associated with older age.

We did not find a significantly increased mortality risk associated with any thoracic injuries investigated in our multivariate logistic regression. While this has been reported before by Jung et al. in their multivariate logistic regression for subcutaneous emphysema and hemothorax, they stated hemopneumothorax and cardiac contusion as significant predictors of mortality in pediatric thoracic trauma [15]. 

While Meier et al. published higher mortality rates in their multiply injured pediatric collective (0- to 5-year-olds 29%, 6- to 12-year-olds 13%, 13- to 17-year-olds 28%) they found injuries to the head, abdomen or thoracic region associated with the highest overall mortality [24]. In further literature, patients with chest injuries showed a mortality rate of 5%, rising to 40% when abdominal or head injuries were included [25, 39]. In contrast, our multivariate logistic regression analysis did not reveal a significantly increased mortality risk with additional injuries (AIS ≥ 3) to the head, abdomen or extremities. Here, one must keep in mind our specially designed cohort consisting of children and adolescents with at least one serious injury to the chest (AIS_Thorax_ ≥3). But we were able to show a significant independent mortality risk with at least one severe injury (AIS ≥ 4) to any body region. Additionally, we report a significant 3-fold increase in mortality for polytraumatized patients, as defined by the “Berlin definition”, which is a patient defined with significant injuries (AIS ≥ 3) in two or more different anatomic AIS regions in conjunction with at least one defined pathophysiologic parameter [21]. This highlights the fact that not only diagnoses of injuries, but even more so do the individual response to trauma indicate the respective injury severity.

It has been published before, that injured children with signs of shock, including hypotension or the need for emergent blood transfusion, have a significantly increased odds of death. And, as a result, these events should signal high acuity and prompt immediate and aggressive resuscitation [11]. This is in line with our finding of a significantly increased independent mortality risk associated with the need for blood transfusion prior to ICU admission. Not surprising is our finding of the highest, 14-fold, independently associated mortality risk, once prehospital cardiopulmonary resuscitation (CPR) had been performed.

Taken together, these findings emphasize that both injury severity and pathophysiologic response mirrored by initial clinical presentation are crucial determinants of survival. Our study confirms, in line with existing literature, that patients suffering from severe chest trauma have a high risk of mortality [1, 15, 26, 37]. The results underscore the importance of standardized trauma management protocols and early identification of high-risk patients to optimize outcomes. Future research should focus on refining early risk stratification tools to guide clinical decision-making and improve survival in pediatric thoracic trauma patients.

### Limitations

There are multiple limitations to this study, mostly due to its retrospective nature. All hospitals participating in the TR-DGU submit to regular audits and sample tests are performed to ensure data quality. However, the documentation’s validity is not controlled by external monitoring as in prospective trials [40]. Although mainly Level-1 and − 2 trauma centers contribute to this database, we cannot comment on locally implemented protocols or specialized training (e.g. simulation training) for trauma care. But our patient cohort received care in German hospitals where training in ATLS^®^ courses and protocols has been established since 2003 [41]. During the years of our study this standardized training spread to participating hospitals, and presumably over time our patient cohort received similar early clinical treatment according to their injury severity. Nevertheless, contributing hospitals to the database change over time and so does the mixture of participating trauma center levels. Additionally, pediatric trauma patients are treated in Level-1, -2 and − 3 trauma centers, but our analysis only includes data documented by the extensive standard data collection form, thus bias is potentially shifting the distribution to higher hospital treatment levels.

Entering data into a standardized platform and their interpretation has limitations regarding age-dependent physiological values. In our study SBP ≤ 90mmHg was defined as shock, but infants have a physiologic SBP of 90mmHg, thus the definition of shock in this youngest population is distorted. On the other hand, we still report a blood transfusion rate in our youngest population (0–5 years) of 18.6%, which might serve as a surrogate parameter for hemorrhagic shock in this age group, as well as a rate of prehospital catecholamine administration in 14.7%. The individual indication for thoracic surgery in our collective remains elusive, since this documentation cannot be found in the database. Additionally, documentation of specific surgical interventions changed in the year 2020 for the entire database, therefore we cannot comment on specific thoracic surgery because this parameter is inconsistent throughout our evaluated period. Correspondingly, we cannot exclude that e.g. chest drain insertion via mini-thoracotomy was coded as thoracic surgery case.

In our study, we focus on severely injured children with at least serious chest injury (AIS_Thorax_ ≥3). As a result, our findings cannot be readily transferred to severely injured children without serious thoracic injury.

## Conclusions

Treating severely injured children is always challenging, even more so when they have suffered critical trauma to the chest. We have detected significant differences in the mechanism of injury, prehospital and in-hospital treatment between our composed age groups. MAIS ≥ 4 in any body region, polytrauma and need for blood transfusion before ICU admission was independently associated with mortality.

Thus, in this study we offer starting points for age dependent injury prevention and provide information to analyze and question current (pre-)hospital management protocols and team simulation training.

Demographic data and injury mechanisms for different age groups.

Statistical comparison of subgroups was performed for three subgroups (0–16 years) only. Results in young adults (17–20 years) are added for descriptive reasons only.

Mechanism “Traffic” includes injuries sustained during overall activities involving vehicles.

Unaccounted numbers are due to “other accidents”, “unknown” mechanism or missing documentation.

## Data Availability

The data that support the findings of this study are available from the AUC—Academy for Trauma Surgery (AUC—Akademie der Unfallchirurgie GmbH), but restrictions apply to the availability of these data, which were used under license for the current study, and so are not publicly available. Data are however available from the corresponding author J.B. upon reasonable request and with permission of AUC (AUC—Akademie der Unfallchirurgie GmbH, Emil-Riedel-Straße 5, 80538 München, Deutschland).

## Abbreviations

AIS: Abbreviated Injury Scale
ASA: Classification of American Society of Anesthesiologists
AUC: Academy for Trauma Surgery
BE: Base excess
CT: Computed tomography
CPR: Cardiopulmonary resuscitation
DGU: Deutsche Gesellschaft für Unfallchirurgie
ED: Emergency department
FAST: Focused assessment with sonography in trauma
GCS: Glasgow Coma Scale
HEMS: Helicopter emergency medical service
ICU: Intensive care unit
ISS: Injury Severity Score
LOHS: Length of hospital stay
LOICUS: Length of ICU stay
LOMV: Length of mechanical ventilation
MAIS: Maximum AIS
MLR: Multiple logistic regression analysis
MOF: Multiple organ failure
MV: Mechanical ventilation
n: Number of cases
OR: Odds ratio
PRBC: Packed red blood cells
Q: Quartiles
SBP: Systolic blood pressure
SD: Standard deviation
TR-DGU: TraumaRegister DGU^®^
TXA: Tranexamic acid
WB-CT: Whole-body computed tomography
